# Benzodiazepine prescribing behaviour and attitudes: a survey among general practitioners practicing in northern Thailand

**DOI:** 10.1186/1471-2296-6-27

**Published:** 2005-06-23

**Authors:** Manit Srisurapanont, Paul Garner, Julia Critchley, Nahathai Wongpakaran

**Affiliations:** 1 Department of Psychiatry, Faculty of Medicine, Chiang Mai University, Amphur Muang, Chiang Mai, 50200 Thailand; 2 International Health Research Group, Liverpool School of Tropical Medicine, Pembroke Place, Liverpool L3 5QA UK

## Abstract

**Background:**

Over-prescribing of benzodiazepines appears common in many countries, a better understanding of prescribing practices and attitudes may help develop strategies to reduce prescribing. This study aimed to evaluate benzodiazepine prescribing behaviour and attitudes in general practitioners practising in Chiang Mai and Lampoon, Thailand.

**Methods:**

Questionnaire survey of general practitioners in community hospitals, to estimate: i) use of benzodiazepines for anxiety/insomnia, panic disorder, depression, essential hypertension, and uncomplicated low back pain and ii) views on the optimal duration of benzodiazepine use.

**Results:**

Fifty-five of 100 general practitioners returned the completed questionnaires. They reported use of benzodiazepines for anxiety/insomnia (n = 51, 93%), panic disorder (n = 43, 78%), depression (n = 26, 43%), essential hypertension (n = 15, 27 %) and uncomplicated low back pain (n = 10, 18%). Twenty-eight general practitioners would prescribe benzodiazepines for non-psychiatric conditions, 17 for use as muscle relaxants. Seventy-five per cent, 62% and 29% of the general practitioners agreed or totally agreed with the use of benzodiazepines for insomnia, anxiety and depression, respectively. Practitioners agreed that prescribing should be less than one week (80%); or from 1 week to 1 month (47%); or 1 to 4 months (16%); or 4 to 6 months (5%) or more than 6 months (2%). Twenty-five general practitioners (45%) accepted that they used benzodiazepines excessively in the past year.

**Conclusion:**

A considerable proportion of general practitioners in Chiang Mai and Lampoon, Thailand inappropriately use benzodiazepines for physical illnesses, especially essential hypertension and uncomplicated low back pain. However, almost half of them thought that they overused benzodiazepines. General practitioner's lack of time, knowledge and skills should be taken into account in improving prescribing behaviour and attitudes.

## Background

Benzodiazepines (BZDs) are approved for treating clinically significant anxiety and insomnia. They have a better safety profile in comparison to barbiturates – their predecessors, and many physicians have overestimated their safety. It is now widely accepted that BZD prescribing has many risks, including tolerance, dependence and misuse, as well as BZD-induced depression, cognitive impairment, disinhibition and psychomotor impairment [1].

BZD has become more restricted recently. The UK data sheets for diazepam and temazepam recommend that BZD should be used for short-term (2 – 4 weeks) management. They are unlikely to be efficacious in the treatment of anxiety after 4 months [2]. BZD addiction can occur when doses within the clinical range are taken regularly over about 6 months [3]. In addition, a recent review suggests that, even in general anxiety disorder – a major indication for BZDs – this treatment may do more harm than good [4].

BZD use has been studied in high income countries, and it appears that prescribing levels have come down as the risks of addiction and adverse effects have become more widely publicised [5]. However, little research has been carried out in developing countries. Limited evidence suggests that BZD use is common in these countries. A recent community survey in Lebanon found that 9.6% (N = 1000) of the population had taken BZD in the previous month [6]. This study also found that approximately 30% of BZD users took these medications for more than 12 months. Although many developing countries allow BZDs to be sold over-the-counter in pharmacies, a literature review of BZD use in Brazil found that the vast majority of BZD consumption was due to medical prescription [7].

While psychiatrists are specialised in caring for people with mental health and substance use problems, their numbers are very limited, especially in developing countries. Therefore, GPs play an important role in prescribing BZDs. For example, a survey in Chile found that 69% of those taking BZDs received the medications from community clinics [8]. A cross-sectional study of prescriptions in 3,368 patients visiting a primary health unit in Brazil also showed that 20.6% of prescriptions included BZDs [9]. A survey of community hospitals in a rural area of Thailand demonstrated that 15% of all adult outpatients received benzodiazepines [10]. These results seem to agree with a previous questionnaire survey among Thai GPs, which found that approximately 50% of them prescribed BZDs for more than 25% of their patients [11]. As there is a trend to increase the provision of mental health care at the primary care level [12], GPs may prescribe more BZDs in the future.

A survey of BZD prescribing practice and attitudes would increase understanding of BZD prescribing problems, important in developing a strategy to reduce BZD prescribing. We therefore proposed to evaluate the prescribing behaviour and attitudes of GPs practicing in Northern Thailand. Two major foci of the survey were the indications for and appropriate duration of BZD treatment.

Because most GPs in developing countries are overloaded by a large number of patients in their everyday practice, patient medical records, e.g. diagnosis, are usually not completed or inaccurate. We therefore decided to conduct a survey using mailed questionnaires.

This survey concerned only the GPs' practice at public settings, where pharmacists take a role in dispensing the prescribed drugs, and GPs practice as non-dispensing physicians. This decision was made because most Thai physicians have their own private practice and are allowed to dispense almost all prescribed drugs, including BZDs, in their private settings. The results of a previous study have shown that dispensing and non-dispensing physicians have different patterns of prescribing [13].

## Methods

### Study population

Questionnaire survey to all 100 general practitioners working in community hospitals located in Chiang Mai and Lampoon, Northern Thailand.

### Questionnaire

The questionnaire for this study included four parts:

i) GP's were asked to say whether they would prescribe BZDs and/or brief (3–5 minutes) supportive psychotherapy/advice for five case vignettes of anxiety/insomnia following a stressful life event, panic disorder, depression, essential hypertension and uncomplicated low back pain (see Annex). Brief supportive psychotherapy/advice could be given by the GPs or other medical professionals in the community hospitals.

ii) GPs were asked whether they agreed with the use of BZDs for clinically significant insomnia, anxiety and depression, as well as non-psychiatric illnesses, by using a 10 cm line of visual analogue scale (VAS) ranging from totally disagree to totally agree. A VAS score of 0.00–1.9, 2.0–3.9, 4.0–6.0, 6.1–8.0, and 8.1–10.0 were classified as totally disagree, disagree, neutral, agree and totally agree, respectively.

iii) the same VAS and its scoring system were used to ask the GPs whether they agreed with the regular use of BZDs for less than 1 week, 1 week to 1 month, more than 1 month to 4 months, more than 4 months to 6 months and more than 6 months.

iv) finally, GPs were asked if they thought they over-prescribed BZDs and if so why (more than one reason could be chosen).

### Procedures

The GPs were informed that the survey did not intend to assess their knowledge but wished to understand their practice. The answers should be based on their everyday practice in public settings only (mainly community hospitals). The GPs were also asked to return the questionnaires with no answer if: i) they did not wish to respond or ii) they saw less than five adult out-patients per week.

To maximize responses, 6 weeks after the first mailing, we sent the questionnaire to all GPs who had not replied. Six weeks after the second mailing, we sent the questionnaire for the third (and last) time to GPs who had still not responded. The survey was carried out between July and November 2003.

### Statistical analysis

Descriptive statistics (percentages, means and standard deviations) were calculated.

## Results

Fifty-eight of 100 GPs (58%) returned the questionnaires to us. Three were excluded; two GPs refused to answer the questionnaire, and another one saw less than five adult patients per week. Questionnaires from 55 GPs (32 males and 23 females) were subsequently analyzed. The GPs mean (SD) age in years was 31.6 (7.1), with a mean of 6.7 (5.8) years in practice and working for an average of 38.7 (18.2) hours per week. The mean number of patients seen per week (SD) was 392.8 (243.9). Of 13 GPs who had special training, four were paediatricians, three were internists, two each were surgeons and orthopaedists, and there was one family practitioner and one obstetrician/gynaecologist.

Table 1 shows the number of GPs giving BZDs and brief supportive psychotherapy/advice for the 5 case vignettes. The majority of GPs would give BZDs for anxiety/insomnia following a stressful life event and panic disorder. The numbers of GPs giving brief supportive psychotherapy/advice for anxiety/insomnia and panic disorder were lower (22% and 15%, respectively). While 47% of the GPs would give BZDs for depression, 27% and 18% would give BZDs for essential hypertension and uncomplicated lower back pain, respectively. Of 26 GPs (47% of the GPs) who gave BZDs for depression, 16 of them would administer antidepressants concurrently.

**Table 1:** Number of GPs giving BZDs and brief supportive psychotherapy or medical advices for the 5 case vignettes (N = 55)

Clinical conditions	No. of GPs (%) giving BZDs	No. of GPs (%) giving brief supportive psychotherapy or advice
1. Stressful life event and anxiety and insomnia	51 (92.7)	39 (70.9)
2. Panic disorder	43 (78.2)	35 (63.6)
3. Depression	26 (47.2)	29 (52.7)
4. Essential hypertension	15 (27.3)	46 (83.6)
5. Uncomplicated low back pain	10 (18.2)	38 (69.1)

Agreement on the indications and durations of BZD use are shown in Table 2. In respect to indications, 75%, 62% and 29% of the GPs agreed or totally agreed with the use of BZD for insomnia, anxiety, and depression, respectively. Twenty-eight GPs specified the use of BZDs for nonpsychiatric illnesses, especially the use of BZDs as muscle relaxants in 17 GPs. Eighty percent, 47%, 16%, 5% and 2% of the GPs agreed or totally agreed with the regular treatment of BZD for less than 1 week, 1 week – 1 month, more than 1 month – 4 month, more than 4 months – 6 months and more than 6 months, respectively.

**Table 2:** Agreement on the use of BZD assessed by using a 10 cm line of visual analogue scale from totally disagree to totally agree*

Issues	Totally disagree, n (%)	Disagree, n (%)	Neutral, n (%)	Agree, n (%)	Totally agree, n (%)
1. For clinically significant insomnia	2 (3.6)	1 (1.8)	11 (20.0)	14 (25.5)	27 (49.1)
2. For clinically significant anxiety	7 (12.7)	4 (7.3)	10 (18.2)	15 (27.3)	19 (34.5)
3. For clinically significant depression	16 (29.1)	11 (20.0)	12 (21.8)	5 (9.1)	11 (20.0)
4. For regular use of less than 1 week	2 (3.6)	1 (1.8)	8 (14.5)	13 (23.6)	31 (56.4)
5. For regular use of 1 week – 1 month	15 (27.3)	2 (3.6)	12 (21.8)	15 (27.3)	11 (20.0)
6. For regular use of more than 1 month – 4 months	28 (50.9)	10 (18.2)	8 (14.5)	5 (9.1)	4 (7.3)
7. For regular use of more than 4 months – 6 months	39 (70.9)	9 (16.4)	4 (7.3)	1 (1.8)	2 (3.6)
8. For regular use of more than 6 months	47 (85.5)	4 (7.3)	3 (5.5)	0 (0.0)	1 (1.8)

*A VAS score of 0.00–1.9, 2.0–3.9, 4.0–6.0, 6.1–8.0, and 8.1–10.0 were classified as totally disagree, disagree, neutral, agree, and totally agree

Twenty-five GPs (45.5%) accepted that they excessively used BZDs in the past year. The reasons for the over-prescribing were lack of time (17 responses), lack of knowledge and skills (14 responses), intention to keep doctor-patient relationship (i.e., patient demand -13 responses), lack of alternative treatment to BZDs (12 responses) and saving costs (10 responses).

## Discussion

This survey was conducted in young Thai GPs practicing in community hospitals. One of the surprising findings was these doctors reported that they would use BZDs for essential hypertension and uncomplicated low back pain, as well as the use as muscle relaxants. Almost half of the GPs agree that they over-prescribe BZDs.

BZD prescribing for essential hypertension and low back pain is relatively common in developing countries [10]. Although there is some evidence supporting the benefits of BZDs for these conditions [14-16], the administration of these drugs may be detrimental [17]. This practice should be obsolete as a number of inexpensive drugs with preferred risk/benefit profiles are widely available, e.g., propanolol, orphenadrine citrate.

The results of this and previous studies [18] demonstrate that BZD prescribing is a dilemma for GPs. Many of them realize the harmful effects of BZDs, but cannot control their prescribing. Improvement of knowledge and skills alone may not solve the problem. GPs in this survey have to see approximately 10 patients per hour, and this time pressure should be taken into account in developing any strategy to solve the problem.

The findings of this survey are helpful in developing a strategy to reduce BZD use, especially among GPs practicing in developing countries. Because almost half of the GPs have already identified BZD prescribing as a problem, a simple and practical strategy to reduce prescribing would be welcomed. As lack of knowledge and skills contributes to the problem, an educational programme should be a part of the strategy. Firm evidence showing that fluoxetine can be used to treat patients with anxiety and/or depression safely and cost-effectively in primary care settings of low-income countries [19] should be presented to the GPs. GPs perceived causes of BZD over-prescribing, e.g., lack of knowledge and skills, lack of alternative treatment to BZDs and saving cost of treatment, could be solved. Improving GPs' communication and training other health professionals, e.g., nurses, to provide brief supportive psychotherapy/advice may be also helpful, especially, in maintaining health professional-patient relationship. In addition, this can be used as an alternative or an adjunct to BZD treatment. Because sufficient consultation time is a key for quality patient care, it should be kept in mind that the impact of any strategy may be reduced if this problem cannot be mitigated.

Some limitations should be considered in interpreting the study findings. First, a questionnaire survey may not reflect the 'real world' practice. As most physicians have realized the detrimental effects of BZDs, the answers of many respondents may be based on their knowledge, or what they perceive to be 'best practice', but not their actual practice. Due to this limitation, we focus our interpretation and discussion only on outstanding findings, e.g., the use of BZDs for physical illnesses, the acceptance of BZD over-prescribing. Second, the moderate response rate (58%) of this survey may have some impact on the validity of this study. Third, the results of present study may not be widely applicable because the GPs in Thailand may have different backgrounds from those in other parts of the world, e.g., culture, education, health care systems. However, the findings may provide insight for further studies elsewhere particularly developing countries. Last, most (16 of 26) GPs who gave BZDs for treating depression would also administer antidepressants concurrently. BZDs and anti-depressants should not be co-prescribed for longer than four weeks [20]. As the survey did not assess duration of BZD co-prescribing in depressed patients, we cannot determine the appropriateness of these co-prescriptions.

## Conclusion

This survey found that a considerable proportion of GPs in Chiang Mai and Lampoon, Thailand inappropriately use BZDs for physical illnesses, especially essential hypertension and uncomplicated low back pain. However, many GPs are aware that they over-prescribe BZDs. The problems of lack of time, knowledge and skills should be taken into account in improving the prescribing behaviour and attitude.

## List of abbreviations

BZD = benzodiazepine

GP = general practitioner

VAS = visual analog scale

## Competing interests

The author(s) declare that they have no competing interests.

## Authors' contributions

MS conceived and initiated the study, conducted the survey and analyzed the data. PG and JC conceived and initiated the study. NW conducted the survey. All authors participated in the writing of successive drafts of the manuscript and all have read and approved the final manuscript.

## Pre-publication history

The pre-publication history for this paper can be accessed here:



## Supplementary Material

Additional File 1 Five case vignettes used in the survey. Click here for file
