# Supplementary material for: Benzodiazepine prescribing behaviour and attitudes: a survey among general practitioners practicing in northern Thailand
Source: BMC Fam Pract. 2005 Jun 23;6:27. doi: 10.1186/1471-2296-6-27 (PMC1182364; doi:10.1186/1471-2296-6-27)
Supplement: Additional File 1 — Five case vignettes used in the survey. [file 1471-2296-6-27-S1.pdf]

Five case vignettes used in the survey

Case 1: Anxiety/insomnia following stressful life event

Mr. A is a 28-year old man who works as a foreman in a factory complains to you that he has had an anxious mood for 1 week. At the same time, he has to spend about an hour or two in bed before he can fall asleep. These symptoms started 1 week ago after he found that he could not finish his work by the deadline of a small contract. His wife informs you that the delay of his work affect very little with Mr. A's work. Mr. A has no other physical or mental health symptoms and can function as usual. Mr. A also knows that his anxious mood and insomnia are caused by the stressful life event.

Case 2: Panic disorder

Mrs. B is a 25-year old housewife who complains to you that she has had about 10 episodes of palpitations, sensations of shortness of breath, sweating and feeling dizzy over the past month. Each episode lasts for 30 – 60 minutes, causes her frightened and interrupts what she is doing. She accepts that she cannot get along well with her husband for almost a year, and they started discussing about the divorce 2 months ago. She realizes that her physical complaint is more or less related to the relationship problem. The physical examination and basic laboratory tests reveal no physical problem.

Case 3: Depression

Mrs. C is a 32-year old widow who works as a teacher. She complains you about her loss of appetite and insomnia over the past few months. She also has depressed mood and suicide idea during that period. Over the past month, the symptoms seem to be worse. She has had those symptoms almost all of the times and has not been able to

go to work. She has no stressful live event. The physical examination and basic laboratory tests reveal no physical problem.

Case 4: Essential hypertension

Mr. D is a 40-year old businessman who complains you that he has generalized headache for a few months. A few months ago, he had a physical check-up and found that his blood pressure was 150/100 mmHg. For this visit, his blood pressure is still 150/100 mmHg even after a 10-minute rest. The physical examination and basic laboratory tests reveal no other physical problem.

Case 5: Uncomplicated low back pain

Mr. E is a 38-year old labourer who complains you that he has backache for 3 days. This problem occurred after he worked harder than normal for 5 consecutive days. After a complete physical examination, you find that he has no other physical or neurological signs, except the spasm of his back muscles.
